# High throughput cross-interaction measures for human IgG1 antibodies correlate with clearance rates in mice

**DOI:** 10.1080/19420862.2015.1043503

**Published:** 2015-06-05

**Authors:** Ryan L Kelly, Tingwan Sun, Tushar Jain, Isabelle Caffry, Yao Yu, Yuan Cao, Heather Lynaugh, Michael Brown, Maximiliano Vásquez, K Dane Wittrup, Yingda Xu

**Affiliations:** 1 Department of Biological Engineering; Koch Institute for Integrative Cancer Research; Massachusetts Institute of Technology; Cambridge, MA, USA; 2 Protein Analytics; Adimab; Lebanon, NH, USA; 3 Computational Biology; Adimab; Palo Alto, CA, USA; 4 Department of Chemical Engineering; Koch Institute for Integrative Cancer Research; Massachusetts Institute of Technology; Cambridge, MA, USA

**Keywords:** high throughput screening, developability, monoclonal antibody, PK, clearance, cross-interaction, self-interaction, non-specificity, stickiness

## Abstract

Although improvements in technology for the isolation of potential therapeutic antibodies have made the process increasingly predictable, the development of biologically active monoclonal antibodies (mAbs) into drugs can often be impeded by developability issues such as poor expression, solubility, and promiscuous cross-reactivity. Establishing early stage developability screening assays capable of predicting late stage behavior is therefore of high value to minimize development risks. Toward this goal, we selected a panel of 16 monoclonal antibodies (mAbs) representing different developability profiles, in terms of self- and cross-interaction propensity, and examined their downstream behavior from expression titer to accelerated stability and pharmacokinetics in mice. Clearance rates showed significant rank-order correlations to 2 cross-interaction related assays, with the closest correlation to a non-specificity assay on the surface of yeast. Additionally, 2 self-association assays correlated with each other but not to mouse clearance rate. This case study suggests that combining assays capable of high throughput screening of self- and cross-interaction early in the discovery stage could significantly lower downstream development risks.

## Abbreviations

mAb: monoclonal antibody
PSR: poly specificity reagent
SMP: soluble membrane proteins
CSI-BLI: clone self-interaction-biolayer interferometry
CIC: cross-interaction chromatography
SEC: size exclusion chromatography
AC-SINS: affinity capture self-interaction nanoparticle spectroscopy
SEC: size exclusion chromatography

## Introduction

Development of a lead antibody candidate into a therapeutic drug is a long, expensive, and risky process. Many monoclonal antibody (mAb) candidates have failed development ultimately due to lack of drug-like biophysical properties, i.e., poor expression and manufacturability, low stability and solubility, high viscosity and fast serum clearance. To minimize downstream risks, predictive assays have been developed and applied to screen for desirable biophysical properties early in the development process prior to moving the lead molecule(s) forward. Among these assays, some are designed to identify mAbs that cross-interact with a diversified population of proteins distinct from their specific binding targets. For example, cross-interaction chromatography (CIC) is designed to test weak cross-interaction of a mAb when flowing through a column coupled with human serum polyclonal antibodies.^1^ Late elution is indicative of exposure of interaction-prone surfaces in the mAb that are capable of non-specific binding, a result often linked to poor solubility. A similar ELISA-based approach uses the membrane proteins presented on the surface of a baculovirus particle (BVP) as a reagent to capture mAbs with cross-interaction propensity.^2^ BVP binding correlates well with faster serum clearance.^2^ Similarly, soluble membrane proteins (SMP) have been developed primarily as an early discovery stage polyspecificity reagent (PSR), enabling negative sorting during in vitro mAb selection to guide the binding population away from non-specificity, or to perform post-selection characterization for individual mAbs.^3^

Complementary assays have also been devised to measure mAb self-interaction. Due to the low binding affinities of self-interaction between Fab:Fab or Fab:Fc, either sensitive analytical tools or high concentrations of mAbs are necessary to directly observe mAb self-association. For example, BIACORE^4^ and Biolayer Interferometry (BLI)^5^ have been used for real-time observation of self-association and dissociation of mAbs. Self-binding responses observed by clone self-interaction BLI (CSI-BLI) correlate well with HPLC-based self-interaction chromatography retention times. However, the BLI-based self-binding assay can be done in a high throughput manner with much less material consumption. Alternatively, direct observation of mAb self-interaction is enabled by gold nanoparticles using self-interaction nanoparticle spectroscopy (SINS).^6-8^ The mAb of interest is loaded directly^9^ or through capturing antibodies (Affinity capture SINS, AC-SINS),^10^ to the surface of gold nanoparticles. The mAbs prone to self-association cause clustering of nanoparticles, which can be monitored by plasmon wavelength shift. These assays targeting mAb self-interaction are useful tools during formulation screening for best buffer composition to minimize mAb self-interaction, which is important during development of formulations for subcutaneous administration and mAb storage condition scouting for longer shelf life.

Here we report a case study of 16 mAbs found previously to exhibit varying degrees of self- and cross-interaction as assessed by a panel of high-throughput assays. Their downstream behaviors such as expression titer, aggregation propensity and mouse serum clearance show interesting correlations with these assay predictions, with pharmacokinetics (PK) correlated to cross-interactions metrics, and self-interaction assays forming a self-correlated cluster.

## Results

### Measurement of clearance rates in mice for a panel of human IgG1 antibodies

Sixteen fully human or humanized IgG1 mAbs against multiple targets were either discovered at Adimab (n = 12) or expressed recombinantly from published variable region sequences (fully human: ganitumab,^11^ olaratumab;^11^ humanized: mepolizumab,^12^ motavizumab^13^). To explore the effect of the variable region in a common context, all of these mAbs were expressed as IgG1 in a HEK 293 cell line and purified by Protein A. This panel was chosen to represent 4 distinct developability profiles based on their early performance in self- and cross-interaction detection assays. Using a cutoff of 500 for PSR MFI and of 5 nm for AC-SINS, we find that 7 mAbs exhibit low levels of both low self- and cross-interaction; 4 mAbs show high self- and cross-interaction; 3 mAbs show high cross-interaction but low self-interaction; and two mAbs show low cross-interaction but high self-interaction. These determinations were guided by previous work using the PSR and AC-SINS assays.^3,6^ Prior to accelerated stability or mouse serum clearance studies, IgG samples with SEC monomer percentage below 90% were subjected to a second step polishing to achieve greater than 90% purity.

**Figure 1.**
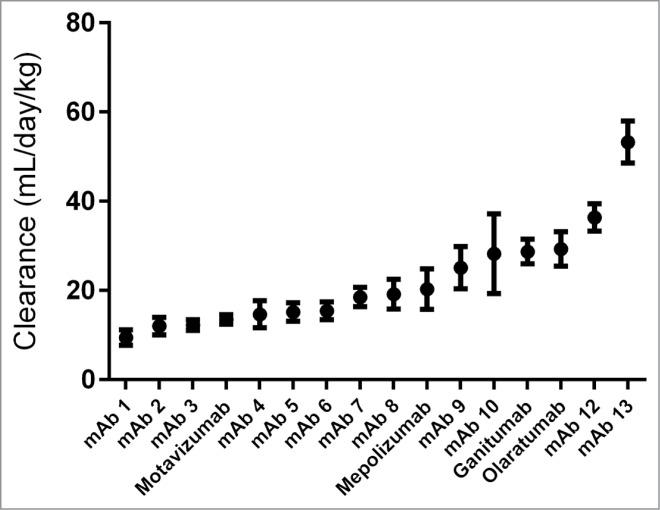
Clearance rates for 16 antibodies in mice. Data from 3 mice per antibody were fit using a biexponential decay model and total clearance was calculated from the combined fit parameters. Error bars represent standard error.

All of the antibodies recognize human- (n = 15) or virus- (n = 1, motavizumab) derived targets, and the absence of mouse cross-reactivity was validated prior to PK evaluation to minimize possibility of target-mediated clearance. The panel displayed a wide range of clearance values (**Fig. 1**), with 7 antibodies having a clearance rate greater than 20 mL/day/kg, a value determined by analysis of receiver operating characteristic (ROC) curves and other considerations (see below) These reported clearance values fall within the expected range for IgG1 clearance rates in mice.^14-16^ The full set of PK curves is displayed in **Figure S1**.

### Correlation of early development tests to mouse clearance rates

**Figure 2.**
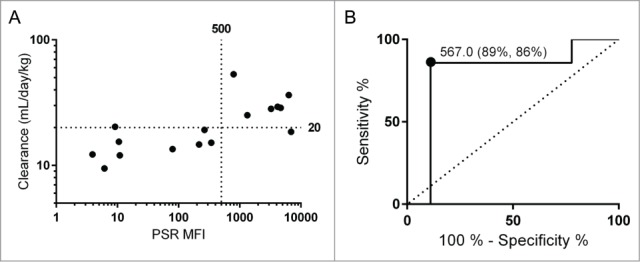
The PSR nonspecificity assay correlates with mouse clearance rates. Clearance rates and PSR scores for the data set are shown with cutoffs of 20 mL/day/kg used for clearance and 500 for PSR MFI (**A**). These cutoffs were determined using an ROC analysis (**B**). For this cutoff, the sensitivity rate is 89% and the specificity rate it 86%, with an area under the curve of 0.79.

Prior to PK studies, the panel of antibodies was subjected to a range of early development assessments. This included PSR median fluorescence intensity (PSR MFI),^3^ AC-SINS Δλ_max_,^6^ CIC retention time,^1^ size exclusion column (SEC) retention time, CSI-BLI response,^5^ accelerated solubility aggregation slope in PBS (% aggregation/day), and purification titer. The full data set is summarized in **Table 1**. Of these assays, 3 showed a significant Spearman rank correlation to mouse clearance rates: PSR binding, CIC retention time, and accelerated stability slope (**Table 2**). Of these 3, clearance rate was correlated most strongly to PSR reagent binding (**Fig. 2A**, Spearman's ρ = 0.72). We additionally performed ROC analysis and Fisher's Exact Test for the PSR assay using a clearance cutoff of 20 mL/day/kg (**Fig. 2B**). This clearance cutoff was chosen based on a combination of allometric scaling and previously reported cutoffs used in cynomolgus monkeys and humans.^2,17,18^ The sum of the true positive and true negative detection rate was maximized at an MFI value of approximately 500. At this threshold, 14 of the 16 antibodies were correctly identified as true positives (n = 6) or true negatives (n = 8), while there was one false positive and one false negative. The associated maximum likelihood for the odds ratio is 48.0 [Fisher's Exact Test, 95% confidence interval (2.47, 933)]. The exclusion of 1 in the confidence interval implies statistical significance, and additionally the standard error in the clearance measurement for each of the false predictions was within the range of the decision boundary.

**Table 1.** Early development tests on a panel of 16 clinical candidate antibodies

Sample	PSR MFI	AC-SINS Δλmax	CSI-BLI response (nm)	CIC RT (min)	SEC RT (min)	Accelerated stability slope (agg%/day)	SEC percent monomer	Purification titer (mg/L)	Clearance (mL/day/kg)
mAb1	6.2	1.1	−0.13	8.4	6.8	0.08	96.3	148.5	9.5
mAb2	10.9	27.9	−0.08	13.7	9.5	1.66	20.9	20	12.0
mAb3	3.9	4.7	−0.11	8.6	6.8	0.11	95.9	117.5	12.2
motavizumab	79.7	3.4	−0.11	8.4	7.1	0.12	95.0	91	13.5
mAb4	216.5	13.3	−0.11	8.8	6.8	0.20	94.9	58.33	14.7
mAb5	342.4	5.5	−0.12	8.8	6.9	0.13	96.1	120	15.2
mAb6	10.6	3.3	−0.13	9.1	6.8	0.13	88.5	170.1	15.4
mAb7	6889.1	1.4	−0.11	20.0	6.8	0.18	90.4	91.67	18.5
mAb8	265.4	6.0	−0.11	9.0	6.9	0.15	95.8	62.67	19.2
mepolizumab	9.2	0.5	−0.14	8.1	6.7	0.10	97.8	110.95	20.3
mAb9	1325.2	29.9	−0.01	10.1	7.8	0.92	69.4	38	25.1
ganitumab	3244.7	27.9	0.28	10.1	6.8	0.20	98.2	79.45	28.2
mAb10	4666.5	2.9	−0.11	20.8	6.8	0.22	88.7	50.83	28.7
olaratumab	4148.4	1.1	−0.13	9.2	6.5	0.61	96.4	109.3	29.3
mAb11	6332.3	28.0	0.18	20.2	7.3	0.28	92.4	110	36.4
mAb12	791.7	8.2	−0.08	9.3	7.6	0.39	69.5	34.17	53.3

**Table 2.** Full correlations of early development assays. Spearman's rank correlations (**A**) and their associated P-values (**B**)

### A minimalist set of early stage assays to predict developability

**Figure 3.**
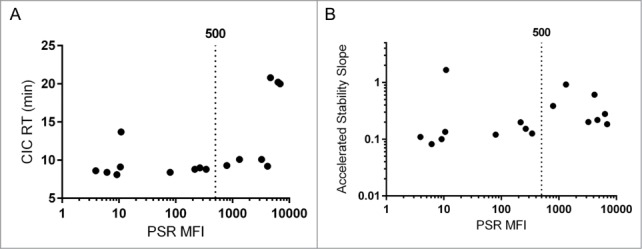
Nonspecificity Assays. The PSR assay correlated significantly with CIC retention time (**A**, Spearman's ρ = 0.79, p value = 0.001) and accelerated antibody stability (**B**, Spearman's ρ = 0.61, p value = 0.015). For each, the used cutoff of 500 for PSR MFI is displayed.

An additional goal of this work was to establish a minimal set of early stage assays to effectively predict developability of a candidate antibody. Toward this goal we have analyzed the correlations and redundancies among the tested metrics. The assays broadly fell into 2 categories, cross- and self-interaction assessment. The two cross-interaction metrics that correlated to clearance (PSR binding, CIC retention time), also correlated with each other (**Table 2**). The relationship between the PSR and CIC assays has been reported prior with a larger set of antibodies^3^ and was confirmed in the new set (**Fig. 3A**, Spearman's ρ = 0.79). Additionally, the PSR assay correlated with accelerated stability (**Fig. 3B**, Spearman's ρ = 0.61), which was less expected as accelerated stability is thought to be a more accurate test of self-interaction as it measures aggregation in a relatively pure preparation.

**Figure 4.**
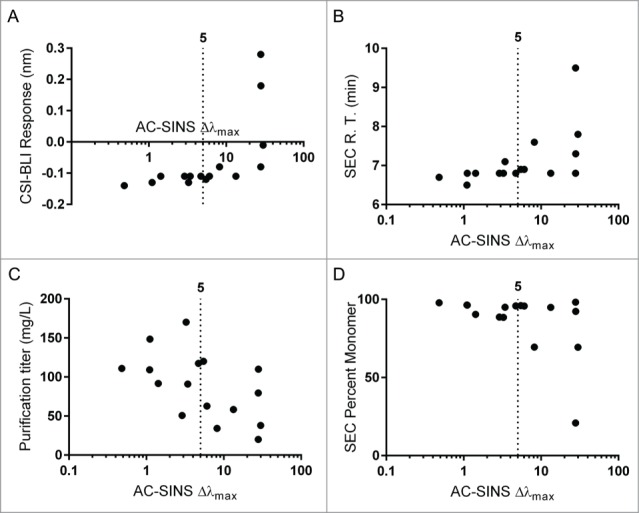
Self Interaction assays. The AC-SINS assay correlated significantly with CSI-BLI response (**A**, Spearman's ρ = 0.87), SEC retention time (**B**, Spearman's ρ = 0.75), purification titer (C, Spearman's ρ = −0.43) and percent monomer post purification (D, Spearman's ρ = 0.52). For each, the cutoff value of 5 nm, suggested by prior work,^6^ is displayed.

The remaining metrics, all related to aggregation propensity, correlated with the AC-SINS assay as well as with each other (**Table 2**). The AC-SINS correlated the strongest to the CSI-BLI assay (**Fig. 4A**, Spearman's ρ = 0.87) as expected, as these 2 assays are identical in all but the format and sensitivity for measurement. The AC-SINS assay also correlated well with relevant antibody production parameters, including SEC retention time (**Fig. 4B**, Spearman's ρ = 0.75), antibody purification titer (**Fig. 4C**, Spearman's ρ = −0.43) and percent monomer after purification (**Fig. 4D**, Spearman's ρ = −0.52).

## Discussion

One of the primary goals of this work was to identify early stage developability assays capable of predicting relative antibody clearance rates of candidate therapeutic antibodies. Previous work found strong correlation between a non-specificity ELISA binding score using baculovirus particles (BVP) and clearance rates in human and cynomolgus monkeys.^2^ In the course of development of the PSR assay used in the current work, PSR was shown to correlate to the baculovirus ELISA.^3^ In this work we have further confirmed that these 2 assays measure related antibody properties, finding that the PSR assay does indeed correlate to clearance rates, in this case in mouse. This is true both using Spearman rank correlation (Spearman's ρ = 0.72, P value = 0.002) as well as Pearson correlation comparing log(PSR MFI) with log(clearance rate) (Pearson's R = 0.73, P value = 0.001). While not formally demonstrating equivalency between the BVP and PSR assays, these results further support the use of PSR binding as an early predictor of antibody developability. (Direct PK measurements in cynomolgus monkeys and humans are beyond the scope of this work.) As an important caveat to the conclusions and correlations presented here, the data set was relatively small (n = 16) and as such any outliers have a strong effect on the calculated correlations.

Clearance rates also correlated to ranking on the CIC assay (**Figure S2A**) and to a lesser extent by the accelerated stability slope (**Figure S2B**). Broadly, CIC and the PSR assay can serve as cross-interaction or specificity metrics. It was not unexpected that the PSR and CIC assays would give similar readouts, as their correlation had been shown prior to this work.^3^ The association of these 2 assays to accelerated stability slope is more unexpected, as there is no obvious explanation as to why a measure of accelerated aggregation propensity should correlate to specificity unless there is an inherent overlap between these 2 mAb characteristics. This correlation was weaker, and may be an artifact of the smaller set of antibodies used in the study. Exploration of this relation in an expanded set of antibodies is the subject of ongoing research in our laboratories and will be reported in due course.

The remaining 5 metrics, AC-SINS, CSI-BLI, SEC retention time, purification titer, and percent monomer, poorly correlated to clearance rates but all showed correlations with each other. These assays fall under the category of self-association or aggregation metrics. The correlation between the AC-SINS and CSI-BLI assays was expected, as they are similar assays and differ only in the format and sensitivity of self-binding. Comparing these 2, the AC-SINS assay is more sensitive than the CSI-BLI assay due to the elevated local concentration of test mAbs at the surface of gold nanoparticles, allowing weak self-interactions to be observed. We did not perform self-interaction chromatography (SIC) on these samples due to material consumption and speed limitation. The correlation to SEC retention time is likely explained by the increase in exposure of non-specific binding interfaces in aggregation-prone antibodies due to colloidal instability. These exposed surfaces interact favorably with the SEC resin, leading to a longer retention time. Lower colloidal stability may also explain the correlation of AC-SINS to both percent monomer and purification titer. The proteins with greater aggregation propensity will be more likely to be degraded in the secretion process by quality control mechanisms, and additionally this will affect the total monomer fraction in the portion that is successfully secreted.

PK of antibodies are largely dictated by interaction with FcRn,^19^ a fact that has been exploited in the past by Fc engineering.^20-22^ However, as the current work and others have shown, antibodies with identical Fc sequences but different variable regions can exhibit disparate PK parameters in wild type mice as well as in other organisms.^23,24^ In particular, the notion that antibodies with poly-reactive^24^ or highly cross-reactive profiles^2^ tend to have faster serum clearance gains further support from the data in this work. While perhaps less appreciated, there are also reports that the interaction of FcRn with antibodies with matched Fc regions but differing variable region sequences could vary.^25,26^ Of particular interest is the observation that the strength of the IgG-FcRn interaction at neutral pH (7.3) correlates negatively with half-life in multiple species and across several series of antibodies.^26^ The weak interactions with FcRn at neutral pH reported in this work might be similar to the nonspecific interactions measured by PSR and BVP.

Additionally, it has been previously reported that a combined metric of high hydrophobicity in certain CDRs or an extreme charge at endosomal pH can be a predictor of clearance in cynomolgus monkey;^27^ however this metric exhibited poor predictive power on the current set using the cutoff of 20 mL/day/kg clearance rate in mice. This metric correctly predicted faster clearance in 1 of 7 mAbs (14%) and normal clearance in 5 of 9 (56%) mAbs (**Figure S3A**). Additionally, as reported before, there was no correlation between antibody pI and clearance rate (**Figure S3B**).

Comparison of the various early development tests reveals 2 major groups of assays with significant internal redundancy, suggesting that selecting one assay from each class would be sufficient as an early indication of antibody developability. Of the 2 groups, cross- and self-association, it is of benefit to select the assay with highest sensitivity and ideally throughput, so it can be applied at the earliest possible stage of antibody discovery. The specificity metrics provide an early prediction of in vivo clearance rates. Of these assays PSR binding is the most appealing, as it is high-throughput and can additionally be utilized as a selection tool for early discovery of developable therapeutic antibodies.^3^ The self-association related metrics provide an early prediction of antibody production and aggregation propensity. From this collection, the AC-SINS assay is the highest throughput, although it cannot be used as a sorting tool. This is true of all of the self-association assays, and highlights a possible value for a rapid self-association assay usable in sorting protocols.

The assays tested here are of sufficient throughput to enable hundreds of measurements in a span of days. Consequently they could be of particular use for screening panels of lead antibodies at a reasonably early stage in the discovery process, and could assist in narrowing the field to 10 or fewer lead molecules for greater scrutiny.

## Materials and Methods

### Determination of clearance in mice

Antibody PK data were determined in C57BL/6 mice using a single bolus intravenous dose. Each antibody was first labeled with Alexa Fluor 647 NHS Ester dye (Life Technologies #A37573), and free dye was removed using a Zeba desalting column (Thermo #89892). Degree of labeling was verified to be between 0.5 and 2.0 for each antibody using a Nanodrop spectrophotometer (Thermo). Each antibody was dosed via retro-orbital injection at 5 mg/kg into 3 mice. Serum samples were collected immediately after injection and at 0.5, 1, 3, 5, 8, 24, 48, 96, and 168 hours and 2 and 3 weeks post injection. Antibody concentration was determined by measurement of fluorescent intensity using a Typhoon imager after degree of labeling correction (GE Healthcare). Fluorescent measurements were quantified by normalization to a standard curve for each antibody, and PK profiles were fit in Graphpad Prism using a 2 phase non-compartmental model. Fits for the 3 mice in each group were averaged to obtain a single PK curve for each antibody, from which total clearance rate and standard error were calculated. To ensure that labeling of the antibodies had minimal effect on measured clearance rates, PK for one control antibody were measured both with and without label. Non-labeled antibody serum levels were quantified by ELISA using Meso Scale Discovery (MSD).^28^ Calculated clearance rates were near equivalent between labeled (12.95 ± 9.22 mL/day/kg) and unlabeled (14.04 ± 3.49 mL/day/kg) samples suggesting that the fluorescent labeling method replicates similar ELISA based methods (**Figure S4A**).

Additionally, there was some concern of immunogenicity as a result of the injection of human IgG antibodies into immunocompetent mice. To assess this, serum from the 3 mice treated with mAb2, mAb6, and mAb12 or one mouse injected with PBS was collected at the terminal time point and assessed for anti-human IgG1 antibodies. The antibody of interest was first loaded nonspecifically onto 0.2 µM sulfate coated FluoSpheres (Life Technologies #F-8848) by incubation for 30 minutes in PBS. The coated beads were washed once in 1mL PBSA (1x Phosphate Buffered Saline, Corning #21-040-CV, plus 0.1% Bovine Serum Albumin, Sigma #A9418) and then incubated in a 1:100 dilution of the serum in PBSA for 30 minutes. Beads were again washed and positive binding of mouse antibodies was detected via goat anti mouse secondary, AlexaFluor 647 conjugate (Life Technologies # A-21236). Final bead suspensions were washed once more and binding median fluorescence intensity was measured by flow cytometry. These results revealed no significant immunogenic reaction against any of the antibodies over the time course of our measurements (**Figure S4B**).

### Cross-interaction chromatography (CIC)

CIC was performed as described previously.^1^ In brief, the CIC column was prepared by coupling ˜30 mg of human serum polyclonal antibodies (Sigma #I4506) to a 1 mL HiTrap column (GE Healthcare # 17-0716-01), followed by quenching with ethanolamine. Approximately 5 µg of each antibody was tested at a flow rate of 0.1 mL/min using PBS as a mobile phase on an Agilent 1100 series HPLC system.

### Polyspecificity reagent binding assay (PSR MFI)

PSR assay was done as previously described.^3^ In short, soluble membrane proteins were prepared from CHO cells. The enriched membrane fraction was biotinylated using NHS-LC-Biotin (Pierce, Thermo Fisher Cat# 21336). This poly-specificity reagent was incubated with IgG-presenting yeast, followed by washing. Then secondary labeling mix (Extravidin-R-PE, anti-human LC-FITC, and propidium iodide) was added to the mixture. Samples were analyzed on FACSCanto (BD Biosciences) using HTS sample injector. Flow cytometry data were analyzed for median fluorescence intensity in the R-PE channel to assess non-specific binding.

### Clone self-interaction by bio-layer interferometry (CSI-BLI)

CSI-BLI assay was carried out as previous described.^5^ Briefly, human IgG was loaded to AHQ biosensor (ForteBio) to ∼1 nm, followed by sensor blocking with human IgG1 Fc. The self-association was performed at 1 µM solution concentration of IgG for 300 seconds on an Octet HTX system (ForteBio). The binding response from the association step was subtracted from that of a reference IgG (adalimumab). Using the differential signal, instead of absolute signal, allows for the correction of run-to-run variation.

### Affinity-capture self-interaction nanoparticle spectroscopy (AC-SINS)

AC-SINS assay was performed as described previously.^6,10^ In short, gold nanoparticles (Ted Pella Inc. #15705) were coated with 80% capturing anti-human goat IgG Fc (Jackson ImmunoResearch #109-005-098) and 20% with polyclonal goat non-specific antibody (Jackson ImmunoResearch #005-000-003). The antibodies of interest were then incubated with the particles for 2 hours and the wavelength shift was measured using Molecular Devices SpectraMax M2 with SoftMax Pro6 software. The self-interacting clones show a higher wavelength shift away from the PBS sample.

### Accelerated stability size exclusion chromatography slope

Samples were kept 1mg/mL at 40°C for 30 d in PBS (20 mM sodium phosphate, 150 mM sodium chloride, pH 7.4). This buffer was selected as a simple, stringent buffer which aims to mimic physiological conditions. Time points were taken at day 0, 5, 20, and 30, and the samples were then analyzed by SEC (Tosoh Bioscience #0022855). For SEC analysis, the running buffer composition was 200 mM sodium phosphate, 250 mM sodium chloride, pH 7.0. Accelerated stability slope was calculated from the percent aggregated, measured on the SEC.

### Statistical analysis

All statistical analysis was completed with the assistance of Graphpad Prism v. 6.0. While many metrics such as clearance rate and PSR MFI appeared to show normal or log normal distributions, there were many that did not, precluding the use of Pearson's correlations to analyze the full data set. As such, Spearman's rank correlations were calculated for all pairwise combinations of antibody characteristics. This notably eliminates the ability to conclude direct correlation between assays, but rather conclude correlation between relative rankings on the assays. To assess statistical significance of correlations, and exact P value was calculated for each pairwise comparison.
