# Supplementary material for: High throughput cross-interaction measures for human IgG1 antibodies correlate with clearance rates in mice
Source: MAbs. 2015 Jun 5;7(4):770–7. doi: 10.1080/19420862.2015.1043503 (PMC4622737; doi:10.1080/19420862.2015.1043503)
Supplement: Supplemental_Material.zip [file kmab-07-04-1043503-s001.zip › Supplemental Material.docx]

**Supplementary Figure 1:** Pharmacokinetic measurements for a panel of antibodies. Serum concentration levels for single- bolus intravenous dosing of a panel of clinical and preclinical antibodies were assessed in three mice each. Each time point represents the average serum concentration for the three mice, and these averages were fit for each antibody using a two phase non-compartmental decay curve.

**Supplementary Figure 2**: Correlation of cross interaction assays to clearance. The clearance rate in mice correlated with CIC retention time (A, Spearman’s ρ = 0.53, p value = 0.036) and long-term antibody stability (B, Spearman’s ρ = 0.52, p value = 0.040). For each, the used cutoff of 500 for PSR MFI is displayed.

**Supplementary Figure 3**: Assessment of prediction power of in silico assays. (A) The in silico developability prediction methods used in Sharma et. al.^27^ were applied to the current data set using a cutoff of 20 mL/day/kg as the dividing line between high (red points) and low (green points) clearance rate. Using the suggested decision boundaries given in the original work, there was little predictive power to the method, with correctly predicted faster clearance in 1 of 7 mAbs (14%) and normal clearance in 5 of 9 (56%) mAbs. (B) Antibody pI for the variable region alone was evaluated computationally for the full set of antibodies and compared to clearance rate in mice. There was no significant correlation found between pI and clearance rate (Spearman’s ρ = -0.13, P value = 0.615). For a given variable region sequence, the net charge at a given pH and the pI were calculated by adding up contributions from ionizable groups using t pKas as given in EMBOSS (<http://emboss.sourceforge.net/apps/release/6.6/emboss/apps/iep.html>)and the Henderson–Hasselbalch equation.

**Supplementary Figure 4**: Validation of pharmacokinetic measurement methods. (A) Pharmacokinetic measurements were made for both labeled and unlabeled variants of a control antibody and fit as in other cases. There was no significant difference between the labeled (circles, solid line, 12.95 ± 9.22 mL/day/kg clearance rate) and unlabeled (triangles, dotted line, 14.04 ± 3.49 mL/day/kg) samples suggesting that the fluorescent labeling method replicates similar ELISA based methods. (B) Immunogenicity of three antibodies was assessed by a bead binding assay. Loading of the antibody of interest was validated by binding of a goat anti human secondary (Ab Loading) and compared against responses to secondary alone (Secondary), serum from PBS treated mice (PBS), or serum from antibody treated mice (Treated). No anti-human IgG antibodies were detected in any of the three cases.
