# Supplementary material for: High throughput cross-interaction measures for human IgG1 antibodies correlate with clearance rates in mice
Source: MAbs. 2015 Jun 5;7(4):770–7. doi: 10.1080/19420862.2015.1043503 (PMC4622737; doi:10.1080/19420862.2015.1043503)

Supplementary Figure 1

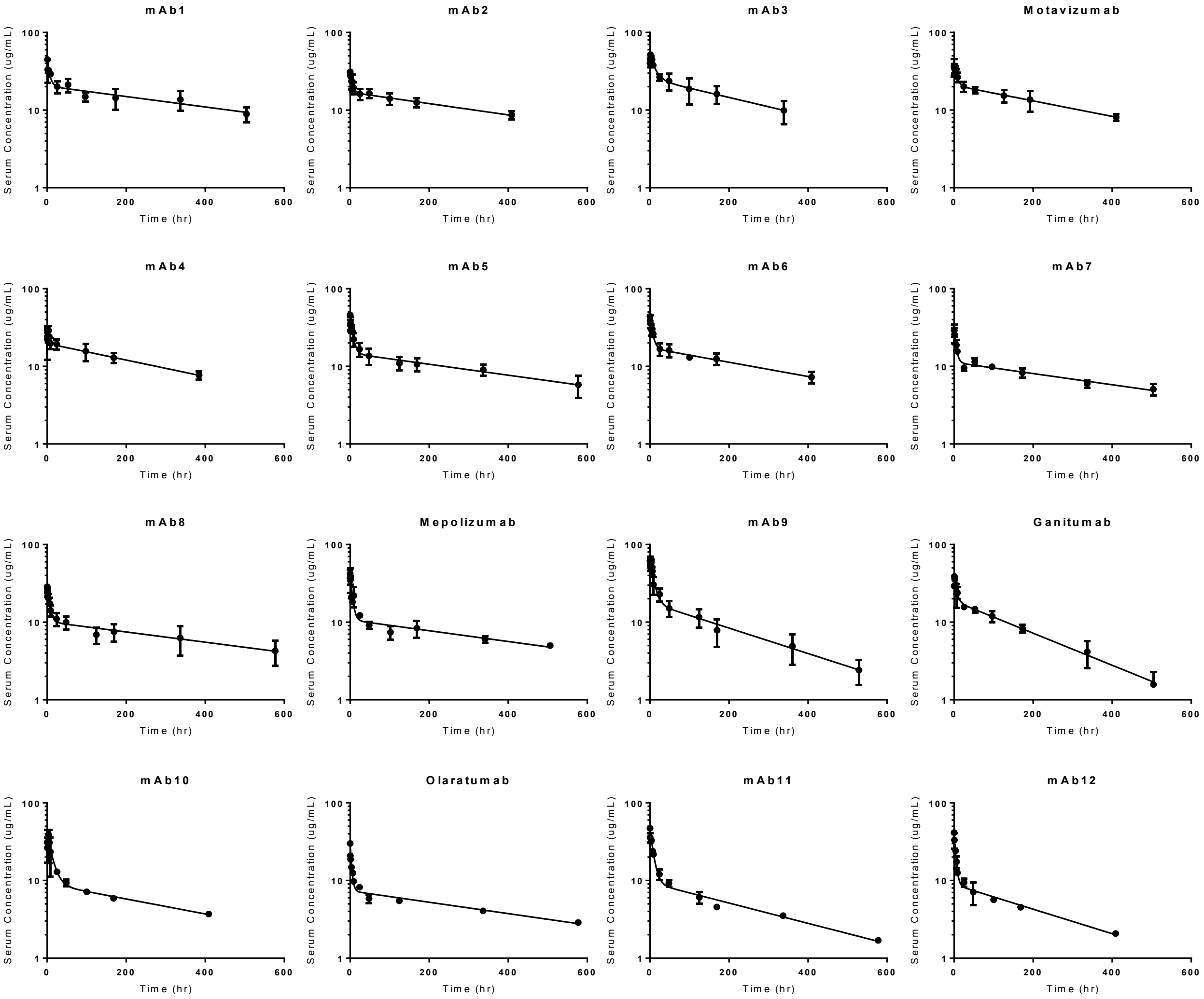

Supplementary Figure 2

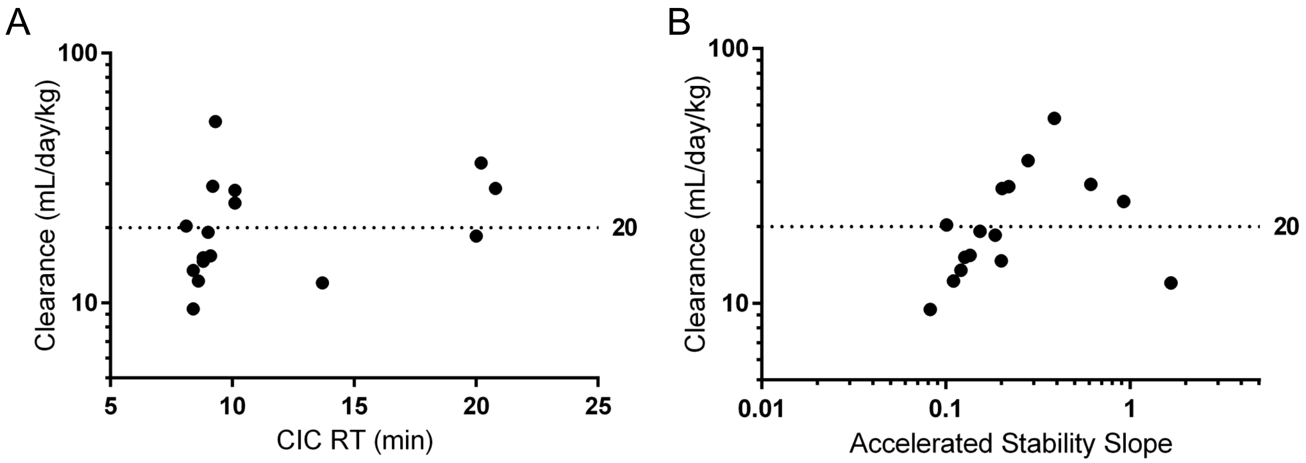

Supplementary Figure 3

A

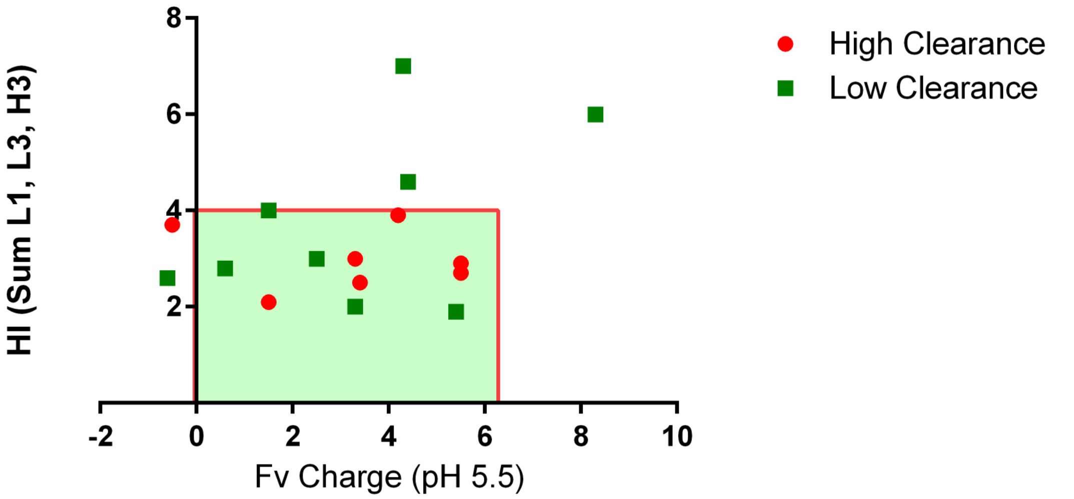

B

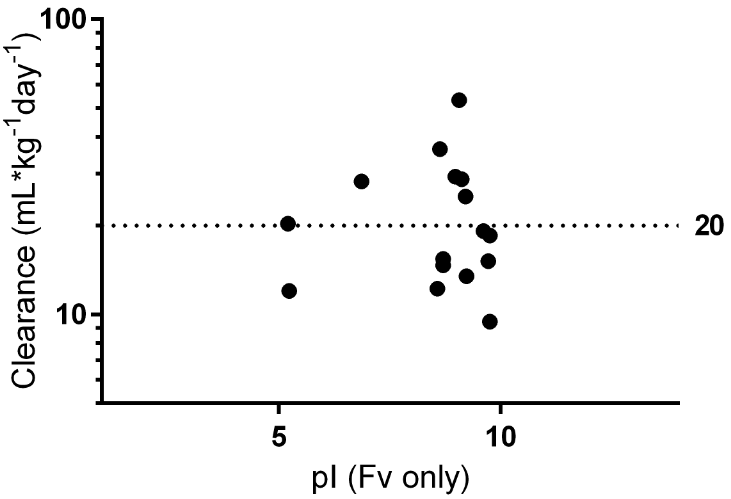

Supplementary Figure 4

A

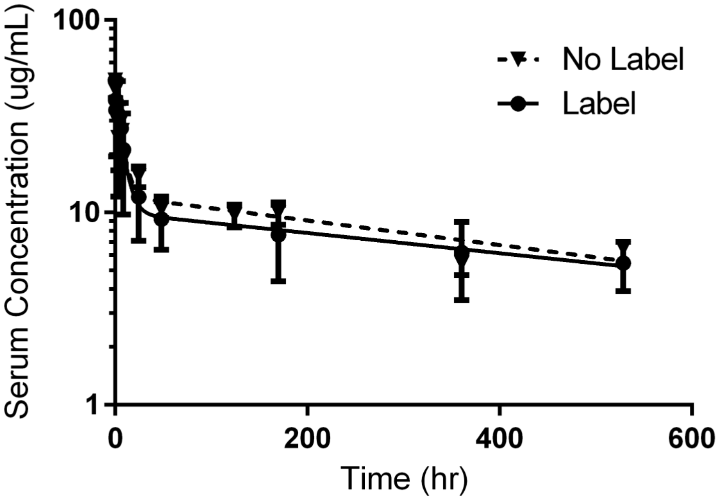

B

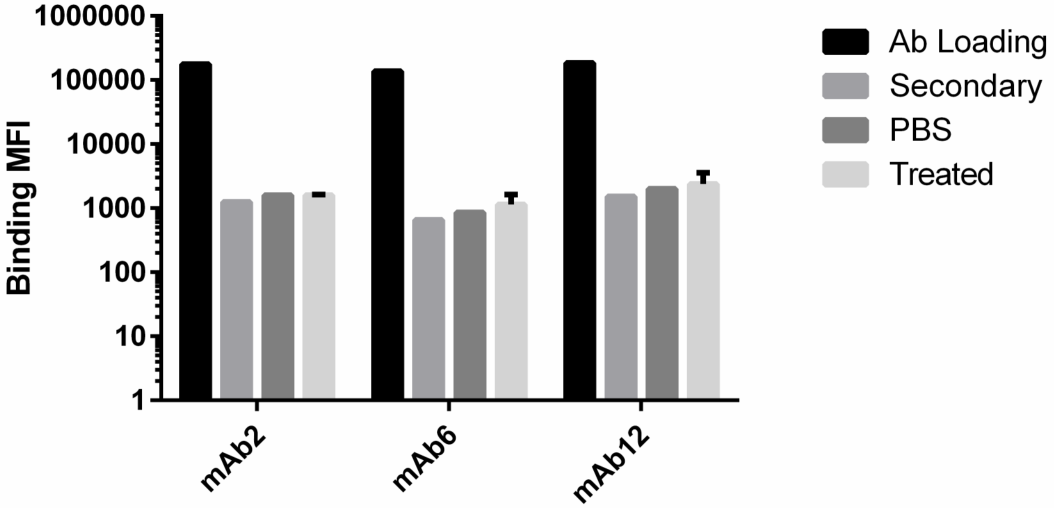

Supplement: Supplemental_Material.zip [file kmab-07-04-1043503-s001.zip › Supplemental figures.pdf]
